# Echoes of Scleroderma: A Case Report Unmasking CREST (Calcinosis, Raynaud's Phenomenon, Oesophageal Dysmotility, Sclerodactyly, and Telangiectasia) Syndrome With Point-of-Care Ultrasound in Acute Medicine

**DOI:** 10.7759/cureus.95671

**Published:** 2025-10-29

**Authors:** Noor Un Nahar, Paola Lopez, Farhaan Ahmed, Yewande Adegeye, Florina Stanley

**Affiliations:** 1 Acute Internal Medicine, Northampton General Hospital, Northampton, GBR; 2 Emergency Medicine, Northampton General Hospital, Northampton, GBR; 3 Internal Medicine, University Hospitals Birmingham NHS Foundation Trust, Birmingham, GBR; 4 Internal Medicine, Northampton General Hospital, Northampton, GBR

**Keywords:** clinical examination, limited cutaneous systemic sclerosis, missed diagnosis, point-of-care ultrasound (pocus), pulmonary hypertension

## Abstract

CREST syndrome, a limited form of systemic sclerosis (SSc), is characterised by calcinosis, Raynaud's phenomenon, oesophageal dysmotility, sclerodactyly, and telangiectasia. It is an autoimmune disorder marked by connective tissue fibrosis and vasculopathy, with pulmonary arterial hypertension (PAH) being one of its most severe complications. PAH has a poor prognosis and often presents with nonspecific symptoms, which can delay diagnosis and treatment.

We present the case of a 54-year-old woman with progressive shortness of breath and bilateral leg swelling, initially managed as possible heart failure. Imaging ruled out pulmonary embolism, but further review revealed oesophageal fluid. Clinical examination showed peri-oral puckering, telangiectasia, calcinosis, and sclerodactyly, raising the suspicion of CREST syndrome. Bedside echocardiography suggested pulmonary hypertension, while serology confirmed strongly positive antinuclear and anticentromere antibodies. She was referred to rheumatology and cardiology, with plans for mycophenolate mofetil initiation and further PAH-focused management.

This case highlights the diagnostic challenges associated with CREST syndrome and emphasises the importance of thorough clinical assessment, imaging review, and antibody testing. Early identification of SSc and its complications allows for prompt intervention, multidisciplinary management, and better patient outcomes in conditions that typically have a poor prognosis.

## Introduction

CREST syndrome, comprising calcinosis, Raynaud's phenomenon, oesophageal dysmotility, sclerodactyly, and telangiectasia, falls under one of the two types of systemic sclerosis (SSc), which are autoimmune chronic fibrosis of connective tissues and vasculopathy leading to the scarring of multiple organs, hence presenting as a constellation of symptoms [1]. One of the most serious complications of SSc is pulmonary hypertension, with a prevalence of four in 1000 cases of limited SSc as per a study conducted in a French cohort and an overall incidence of 6.1 in 1000 cases of SSc [2]. Management and screening of pulmonary arterial hypertension (PAH) is a crucial step, as there is a 55% one-year survival rate as per a French-based study [3].

PAH is defined as a mean pulmonary arterial pressure of >25 mmHg and a pulmonary capillary wedge pressure of <15 mmHg on right heart catheterisation. The presentation of PAH varies, with nonspecific symptoms (dyspnoea, fatigue, peripheral oedema, palpitations, syncope, stress-induced vomiting, and chest pain) causing a delay in diagnosis [4,5]. Treatment options include endothelin receptor antagonists, prostacyclin analogues, phosphodiesterase-5 (PDE5) inhibitors, and guanylate cyclase stimulators. There are further studies being conducted on the use of anticoagulation [2]. 

In view of the importance of early diagnosis, we present a case where these nonspecific symptoms led to the diagnosis of CREST syndrome, with the hope that it aids medical practitioners in its prompt diagnosis, keeping in mind a wide range of differential diagnoses. 

## Case presentation

**Figure 1:**
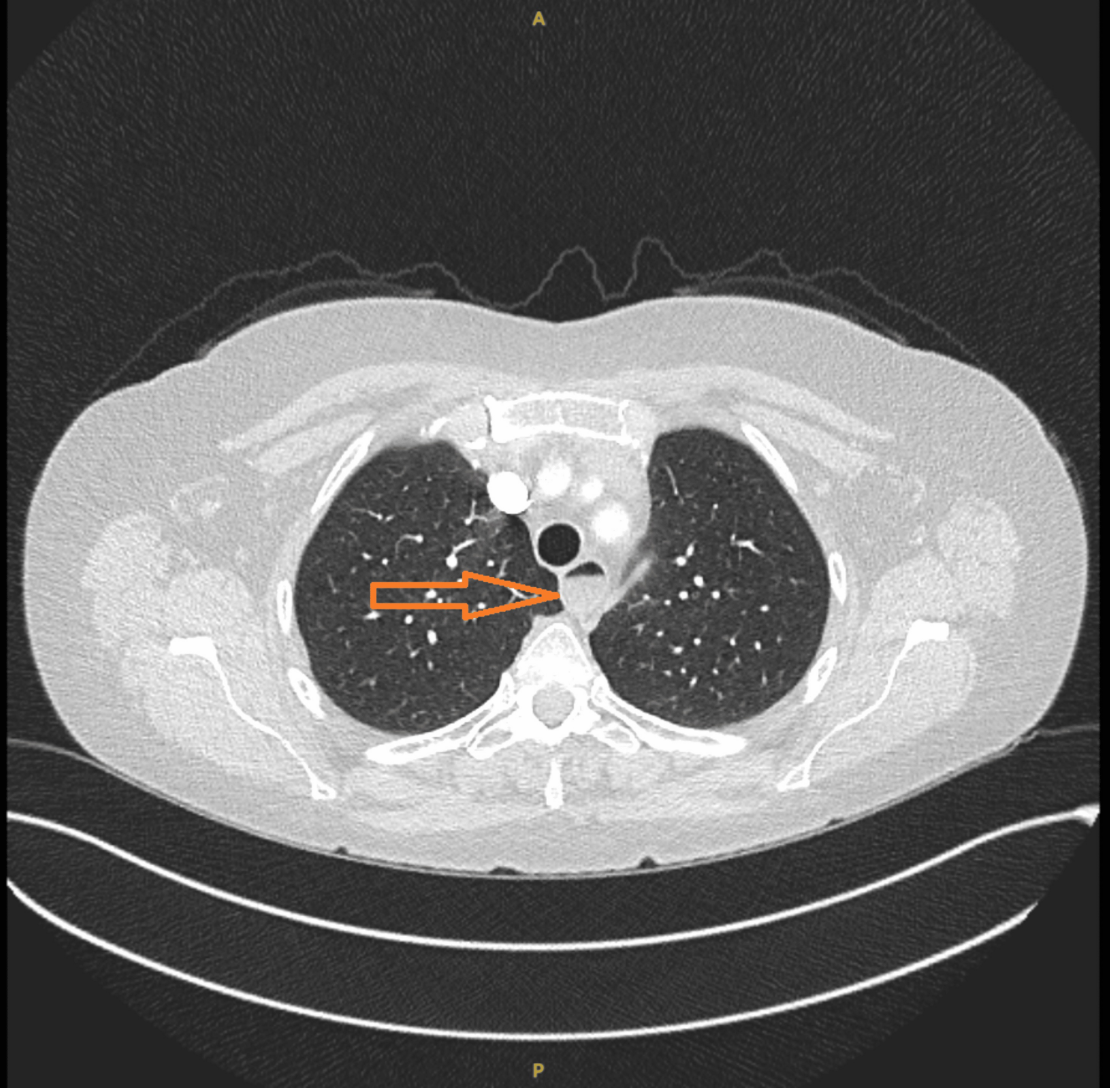
Axial contrast-enhanced CTPA showing normal-calibre pulmonary arteries with no filling defects. Mild oesophageal dilatation with retained fluid is noted CTPA: computed tomography pulmonary angiography

A 54-year-old Caucasian lady presented with a three-month history of worsening shortness of breath and bilateral leg swelling. These symptoms were initially thought to be secondary to heart failure, for which she had an N-terminal pro B-type natriuretic peptide (NT-proBNP) test and an outpatient echocardiography requested. Given the severity of her shortness of breath, which was limiting her mobility, a computed tomography pulmonary angiography (CTPA) was performed to rule out a pulmonary embolism. Figure 1 shows the relevant image.

As the CTPA was negative for pulmonary embolism, she was discharged on furosemide, and a same-day emergency care (SDEC) outpatient referral was arranged to follow up on her symptoms within one week. She later presented to SDEC for a review, where she reported no improvement in her symptoms, and we proceeded to obtain a detailed history. She reported worsening exertional shortness of breath over three months and persistent bilateral ankle swelling. Additionally, she complained of hair loss, burning chest symptoms, joint pain, tightening of the skin on her hands, fingers turning blue in cold weather, and general fatigue. There was no history of oral lesions, dysphagia, cough, chest pain, altered bowel habits, or urinary symptoms. 

The patient had a past medical history of pleomorphic adenoma, oesophageal candidiasis, endometrial benign polyp, solitary left kidney, and excess alcohol intake. There was a regular prescription of omeprazole 20 mg per oral once in the morning. She had a 25-pack-year history of smoking cigarettes but currently vapes. There was no history of recreational drug use. 

**Figure 2:**
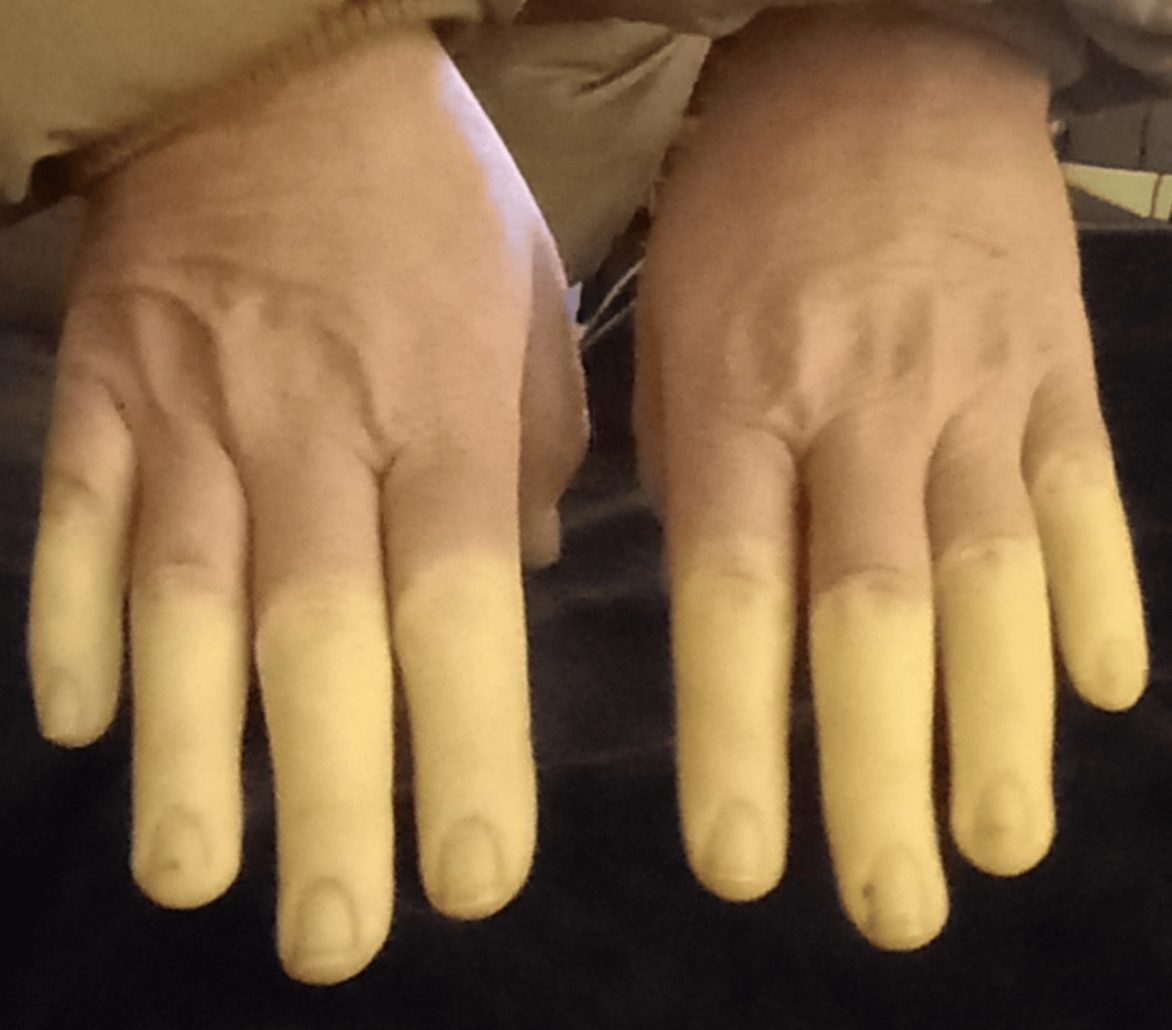
Clinical photograph demonstrating digital sclerodactyly and Raynaud's phenomenon

On examination, she exhibited peri-oral puckering and dry mouth, along with one telangiectasia on the right hand and two on the left hand, grade 3 sclerodactyly of the distal to proximal interphalangeal (PIP) joints, and thickening and calcinosis of the hands bilaterally. There were prominent venules on the anterior aspect of the legs; the rest of the examination systemically was unremarkable. Figure 2 presents a photograph of the patient's hands demonstrating significant clinical findings. 

Her set of baseline blood tests (Table 1) showed raised NT-proBNP.

**Table 1:** Baseline investigations Hb: hemoglobin; WBC: white blood cell; Plt: platelet; MCV: mean cell volume; CRP: C-reactive protein; eGFR: estimated glomerular filtration rate; Na⁺: sodium; K⁺: potassium; ALT: alanine aminotransferase; TSH: thyroid-stimulating hormone; NT-proBNP: N-terminal pro B-type natriuretic peptide

Test	Result	Reference range	Interpretation
Hb	139 g/L	120-160 g/L	Normal
WBC	5.5×10⁹/L	4.0-11.0×10⁹/L	Normal
Plt	318×10⁹/L	150-400×10⁹/L	Normal
MCV	98 fL	80-100 fL	Normal
CRP	5 mg/L	<5 mg/L	Negative
Urea	3.5 mmol/L	2.5-7.8 mmol/L	Normal
eGFR	86 mL/min/1.73 m²	>90 mL/min/1.73 m²	Normal renal function
Creatinine	78 µmol/L	60-110 µmol/L	Normal
Na⁺	138 mmol/L	135-145 mmol/L	Normal
K⁺	3.5 mmol/L	3.5-5.0 mmol/L	Lower limit of normal
Albumin	39 g/L	35-50 g/L	Normal
Calcium (corrected)	2.25 mmol/L	2.2-2.6 mmol/L	Normal
ALT	<10 U/L	0-35 U/L	Low-normal; not clinically significant
Troponin	<13 ng/L	<14 ng/L	Normal (no acute myocardial injury)
TSH	2.0 mIU/L	0.4-4.0 mIU/L	Normal thyroid function
D-dimer	600 ng/mL FEU	<500 ng/mL FEU	Negative for age
NT-proBNP	3963 pg/mL	<125 pg/mL	Significantly elevated; suggests possible heart failure

**Figure 3:**
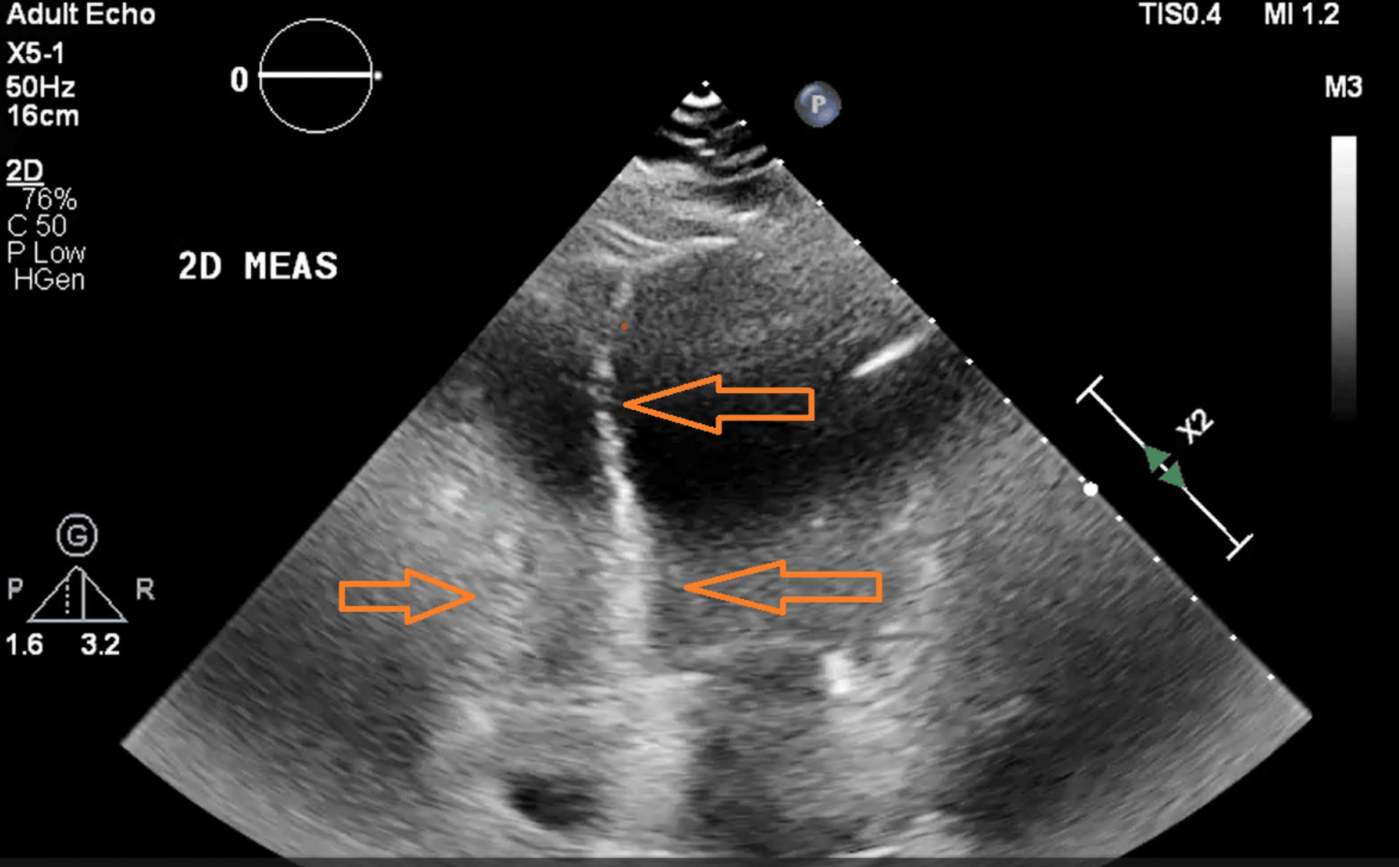
Four-chamber view demonstrating a moderately dilated right ventricle and right ventricle hypertrophy of 9.4 mm with an elevated right ventricular systolic pressure at 40-50 mmHg, consistent with pulmonary hypertension. POCUS expedited recognition and referral prior to formal echocardiography POCUS: point-of-care ultrasound

This prompted a bedside point-of-care ultrasound (POCUS) to assess the heart, which indicated a probability of PAH. Figure 3 highlights the findings of POCUS. 

The previously missed findings of free fluid and dilated oesophagus were identified from the formal CTPA report upon review. A detailed clinical examination revealed all the characteristic features of CREST syndrome. A request for autoimmune screening was sent, with antinuclear antibody (ANA) and anticentromere antibodies returning positive. Table 2 presents the detailed autoimmune profile results. 

**Table 2:** Autoimmune profile giving the serological evidence of CREST syndrome Anti-Jo-1 Ab: anti-histidyl-tRNA synthetase antibody; anti-SRP Ab: anti-signal recognition particle antibody; CN-1A Ab: anti-cytosolic 5'-nucleotidase 1A antibody; ANA: antinuclear antibody; ANCA: antineutrophil cytoplasmic antibody; RF: rheumatoid factor; MPO Ab: myeloperoxidase antibody; PR3 Ab: proteinase 3 antibody; anti-dsDNA: anti-double-stranded DNA antibody; CREST: calcinosis, Raynaud's phenomenon, oesophageal dysmotility, sclerodactyly, and telangiectasia

Test	Result	Reference range
Anti-Jo-1 Ab	Negative	Negative
Anti-SRP Ab	Negative	Negative
CN-1A Ab	Positive	Negative
ANA	Centromere pattern	N/A
ANCA	Negative	Negative
C3	1.32 g/L	0.9-1.8 g/L
C4	0.29 g/L	0.1-0.4 g/L
RF	<10 IU/mL	<14 IU/mL
Cardiolipin IgG	8 GPL	<20 GPL
MPO Ab	10 U/mL	<20 U/mL
PR3 Ab	<2.3 U/mL	<20 U/mL
β2 glycoprotein IgM	<1.1 MPL	<20 MPL
β2 glycoprotein IgG	8.7 GPL	<20 GPL
Amyloid pattern	6.2	0-10
Anti-dsDNA	17.1 IU/mL	0-27 IU/mL

She was subsequently referred by our SDEC team to the rheumatology team and PAH clinic with a diagnosis of limited SSc (CREST syndrome) and associated PAH. She is currently being managed by the rheumatology team, who have commenced her on mycophenolate mofetil 500 mg per oral twice daily and hydroxychloroquine 200 mg per oral once daily. She also has pending further investigations. Meanwhile, her repeat NT-proBNP three months after starting the therapy is 89 pg/mL with significant improvement in her symptoms. 

## Discussion

This case highlights a 54-year-old woman presenting with progressive dyspnoea and peripheral oedema, initially suspected to indicate heart failure but ultimately diagnosed as CREST syndrome complicated by PAH. The constellation of Raynaud's phenomenon, sclerodactyly, telangiectasias, calcinosis, systemic fatigue, and positive anticentromere antibodies, which is a highly reliable marker with a sensitivity of 60-80% in quite recent studies, provided the diagnostic clarity [1,6].

Pulmonary hypertension is characterised by a mean pulmonary arterial pressure exceeding 25 mmHg on right heart catheterisation and is marked by increased pulmonary vascular resistance [4]. A study conducted in a French cohort estimated a mean pulmonary arterial pressure in connective tissue disease-associated PAH of 49±17 mmHg, which reflected in our POCUS findings as well [3]. This condition imposes increasing strain on the right ventricle, which initially compensates but eventually fails, leading to worsening dyspnoea, syncope, chest discomfort, peripheral oedema, and a biochemical indication of increased NT-proBNP levels [6,7]. In connective tissue disease-associated PAH, including CREST syndrome, multiple mechanisms act together: chronic autoimmune-mediated endothelial injury promotes fibrosis and vascular remodelling, while endothelial cell dysfunction reduces the production of vasodilators like nitric oxide and prostacyclin [6]. This imbalance encourages vasoconstriction and further remodelling, thus maintaining elevated pulmonary pressures [6,7].

This patient's journey highlights the diagnostic challenges of differentiating PAH from primary cardiac causes. Initial management focused on heart failure, and oesophageal fluid observed on CTPA was attributed to reflux and candidiasis rather than oesophageal dysmotility, a key feature of CREST syndrome [1,6]. This emphasises the importance of correlating incidental imaging findings with systemic features. Bedside evaluation was crucial: focused clinical examination and POCUS identified signs consistent with CREST syndrome and PAH, respectively, leading to a prompt referral to rheumatology and cardiology [7,8]. Echocardiography remains a vital non-invasive tool for assessing pulmonary pressures, although right heart catheterisation remains the gold standard [4,6].

The management of PAH in CREST syndrome is achieved in a number of ways. Amongst the disease-targeted therapies, endothelin receptor antagonists, such as ambrisentan and bosentan, have been proven effective. These drugs reduce the endothelin in the blood and therefore attenuate vasoconstriction. Randomised controlled trials have portrayed the benefits of improvements in functional class, six-minute walking distance (6MWD), and hemodynamic parameters in PAH [9].

PDE5 inhibitors such as sildenafil are also effective in PAH. These inhibitors reduce the catabolism of cyclic guanosine monophosphate (cGMP), potentiating the effects of nitric oxide-mediated vasodilation. In one cohort of patients, sildenafil (20 mg) was shown to have significant improvements in exercise capacity, hemodynamic indices, and functional class. This was achieved after only three months of therapy [9]. Prostacyclin analogues such as treprostinil have demonstrated similar efficacy, but are generally given to patients with advanced disease (New York Heart Association (NYHA) class 4) [10].

This case highlights the importance of early recognition and timely diagnosis in patients with PAH with connective tissue disease. Without treatment, the median survival rate is around one year following diagnosis, and PAH accounts for 30% of deaths in this population. With treatment, the three-year survival of patients with functional capacity type 2 is around 75%. Notably, more than 80% of patients are diagnosed with a functional class of 2 or higher, correlating with a three-year survival closer to 50% on treatment. These studies underscore the importance of having a high index of suspicion of PAH in connective tissue disorders. They also portray how prompt intervention and delayed diagnosis can have significant effects on patients, including mortality and life expectancy [4].

Studies have shown that patients who were screened for PAH with SSc had far greater results than those diagnosed in routine practice. Early screening had significantly greater survival rates at eight years, as opposed to patients diagnosed routinely [11]. It also shows the value of a multidisciplinary approach in diagnosis and management.

Although the patient was initially assessed in the acute medical setting, communication between cardiology and rheumatology was essential. Rheumatology input was crucial in confirming the diagnosis and guiding immunosuppressive therapy, while cardiology referred the patient to our local tertiary centre for pulmonary hypertension management. This integrated approach enabled early intervention. Our patient was commenced on mycophenolate mofetil and hydroxychloroquine and has been referred to our tertiary centre in order to commence PAH-directed therapy.

## Conclusions

This case underscores the critical importance of recognising CREST syndrome as a multisystem disorder, particularly when patients display nonspecific gastrointestinal, dermatological, or cardiopulmonary symptoms. PAH is a severe complication with a poor prognosis, often challenging to detect early due to subtle clinical signs and imaging findings such as mild dyspnoea or oesophageal fluid. A thorough physical examination offers vital diagnostic clues.

Combining serology, echocardiography, detailed imaging, and bedside tools such as POCUS can accelerate diagnosis and risk assessment. Although we were not able to do quantitative measurements with POCUS, subtle signs did point us in the right direction. This case highlights the crucial role of early recognition of PAH in the context of connective tissue disease. Prompt identification allowed early referral to our tertiary centre for further testing and treatment. Following this, the patient can be considered for newer therapies, including endothelial receptor agonists, which may improve long-term outcomes. A challenge in this case was the presentation of SSc in the emergency department. This underlines the need for continued awareness and education to ensure earlier recognition and referral.
